# An Advanced Statistical Approach Using Weighted Linear Regression in Electroanalytical Method Development for Epinephrine, Uric Acid and Ascorbic Acid Determination

**DOI:** 10.3390/s20247056

**Published:** 2020-12-09

**Authors:** David Majer, Tinkara Mastnak, Matjaž Finšgar

**Affiliations:** Faculty of Chemistry and Chemical Engineering, University of Maribor, Smetanova ulica 17, 2000 Maribor, Slovenia; david.majer@um.si (D.M.); tinkara.mastnak@um.si (T.M.)

**Keywords:** epinephrine, adrenaline, ascorbic acid, uric acid, glassy carbon electrode, square-wave voltammetry, weighted linear regression, heteroscedasticity

## Abstract

In this study, the use of weighted linear regression in the development of electrochemical methods for the determination of epinephrine (EP), ascorbic acid (AA), and uric acid (UA) is presented. The measurements were performed using a glassy carbon electrode and square-wave voltammetry (SWV). All electroanalytical methods were validated by determination of the limit of detection, limit of quantification, linear concentration range, accuracy, and precision. The normal distribution of all data sets was checked using the quantile-quantile plot and Kolmogorov-Smirnov statistical tests. The heteroscedasticity of the data was tested using Hartley’s test, Bartlett’s test, Cochran’s C test, and the analysis of residuals. The heteroscedastic behavior was observed with all analytes, justifying the use of weighted linear regression. Six different weighting factors were tested, and the best weighted model was determined using relative percentage error. Such statistical approach improved the regression models by giving greater weight on the values with the smallest error and vice versa. Consequently, accuracy of the analytical results (especially in the lower concentration range) was improved. All methods were successfully used for the determination of these analytes in real samples: EP in an epinephrine auto-injector, AA in a dietary supplement, and UA in human urine. The accuracy and precision of real sample analysis using best weighted model gave satisfactory results with recoveries between 95.21–113.23% and relative standard deviations between 0.85–7.98%. The SWV measurement takes about 40 s, which makes the presented methods for the determination of EP, AA, and UA a promising alternative to chromatographic techniques in terms of speed, analysis, and equipment costs, as the analysis is performed without organic solvents.

## 1. Introduction

Epinephrine (EP), also called adrenaline, is a compound that facilitates neuronal communication. Since EP has several peripheral functions (i.e., functions outside the brain) and is mainly produced by the medulla of the adrenal glands it is classified not only as a neurotransmitter but also as a hormone [1]. Epinephrine plays an important role during physiological stress by enabling a series of actions of the sympathetic nervous system [2]. The human body’s response to epinephrine manifests as increased cardiac output and increased minute ventilation [3]. Due to its physiological effect, EP is used as a standard medicine in emergency healthcare for the treatment of potentially life-threatening conditions such as anaphylaxis, cardiac arrest, severe asthma exacerbation [4], and circulatory shock [5,6,7,8,9,10]. EP is, as shown in this work, an electrochemically active species.

AA and UA both have significant functions in the human body. UA is the final product of purine metabolism. At physiological pH, UA exists mainly as a urate salt [11]. An increased concentration of UA can lead to the formation of monosodium urate crystals (UA calculi). In people with a genetic predisposition, the accumulation of such crystals causes primary gout [12,13,14,15,16]. AA (vitamin C) acts as an antioxidant, provides electrons for crucial enzyme reactions in cells, and is an essential micronutrient in the central nervous system [17,18,19,20].

The standard analytical approaches for the determination of EP are based on chromatographic methods, including high-performance liquid chromatography (HPLC) [21] and gas chromatography coupled with mass spectrometry (GC-MS) [22]. However, these methods are time-consuming, laborious, require expensive instrumentation, and the use of organic solvents. Electrochemical analytical techniques offer an easy to use, rapid, and inexpensive alternative while operating in water-based mediums [23,24].

Validation is an essential requirement that ensures the quality and reliability of the results for all analytical applications [25]. One of the first steps of method validation is the selection of an appropriate calibration model, most often the unweighted linear regression (usually determined using the square of the correlation coefficient *R*^2^). Judging the linearity on the basis of the *R*^2^ alone is not an adequate criterion for accepting the calibration model. This is still too often overlooked in the scientific literature because the calibration curve range is susceptible to the heteroscedasticity of the data (i.e., usually the variance of the measured response increases at a low and/or high concentration of the analyte) [26]. The use of a weighted linear regression with an appropriate weighting factor often lowers the overall error of the method and thus improves the quality of the analytical results [27,28,29,30].

This work presents the use of a weighted linear regression for the validation of the newly developed electroanalytical methods for the determination of EP, AA, and UA using glassy carbon electrode and square-wave voltammetry (SWV). For each analytical method, the limit of detection (LOD), limit of quantitation (LOQ), linearity, accuracy, and precision were determined. The normality distribution was checked by the quantile-quantile (Q-Q) plot and the Kolmogorov-Smirnov (K-S) statistical tests, whereas the homoscedasticity of the data was evaluated by Hartley’s test, Bartlett’s test, Cochran’s C test, and the analysis of residuals. Due to the observed heteroscedastic behavior of the analytical data, a weighted linear regression was used. The accuracy and precision of the methods were verified using the resulting weighted regression model. The developed and validated methods were used for the analysis of real samples by determining the concentrations of EP in an EP auto-injector, AA in a dietary supplement, and UA in human urine. To the best of the authors’ knowledge, such a statistical approach has not yet been used for the electroanalytical method validation of EP, AA, and UA analytes.

## 2. Experimental

### 2.1. Solutions and Reagents

NaH_2_PO_4_ H_2_O (purity > 98%) and Na_2_HPO_4_ 7H_2_O (>99%) were supplied by Acros Organics (Fair Lawn, NJ, USA). KCl, L-(+)-ascorbic acid (for analysis-ISO), and HCl (37%, for analysis-ISO) were supplied by Carlo Erba Reagents (Val de Reuil, France). Potassium hexacyanoferrate(III) (99%) (K_3_[Fe(CN)_6_]) and UA (≥99%) were supplied by Sigma Aldrich (St. Louis, MO, USA). USP standard EP bitartrate (USP) (C_9_H_13_NO_3_ C_4_H_6_O_6_) was supplied by Sigma Aldrich (Rockville, MD, USA). The PBS solution was prepared using ultrapure water (resistivity 18.2 MΩ cm), obtained by means of an ELGA water purification system (Lane End, UK). The solutions of the diluted analyte standards were prepared using 0.15 M phosphate-buffered saline (PBS) with a pH of 6.5, which was purged with nitrogen for 15 min immediately prior to use.

### 2.2. Apparatus

All electrochemical measurements in this study were performed at room temperature using a model PalmSens4 potentiostat/galvanostat, (PalmSens, Houten, The Netherlands), controlled by PSTrace 5.8 (PalmSens) software with a three-electrode system including a glassy carbon electrode (GCE) with a diameter of 3 mm as the working electrode (ItalSens, Houten, The Netherlands), a KCl saturated Ag/AgCl reference electrode (ItalSens), and a Pt wire as a counter electrode (ItalSens). All potentials (*E*) reported in this work refer to the Ag/AgCl(saturated KCl) reference electrode.

Before the electroanalytical measurements were carried out, the surface of the GCE electrode was polished using 0.05 μm Al_2_O_3_ (Buehler, Lake Bluff, IL, USA) to establish the same surface before each series of measurements. The GCE was then thoroughly rinsed with ultrapure water and immersed in an ultrasound bath containing ultrapure water for 2 min. Additional cleaning was performed by the immersion of the GCE in 0.1 M HCl for 5 min under 1.000 V applied *E*. Afterwards, the electrode was thoroughly rinsed with ultrapure water. Water that remained on the electrode was gently wiped with a paper towel without touching the active GCE surface, and the electrode was left to dry at room temperature.

### 2.3. Cyclic Voltammetry

Cyclic voltammetry (CV) was used for two reasons. Firstly, the suitability of the GCE was tested by checking the reversibility of the potassium hexacyanoferrate system (K_3_[Fe(CN)_6_]), which is a reversible and diffusion-controlled reaction. This experiment was carried out in a 1.0 M KCl solution containing 10 mM K_3_[Fe(CN)_6_]. The CV measurement started at an initial *E* of 0.800 V. A potential sweep was performed in the cathodic direction until the switching *E* was reached at −0.300 V. The potential sweep was then reversed towards more positive potentials until the initial *E* was reached. The CV experiment was performed at different sweep rates, i.e., 10, 20, 50, 75, 125, 150, 175, and 200 mV/s. The system needed to pass certain criteria for the reversible diffusion-controlled reaction [31,32]. If these criteria were not reached, the surface of the GCE was polished again, and the CV experiment was repeated until satisfactory results (the criteria) were met. The criteria and results are presented in the Supplementary Material (see Figure S1 in the Supplementary Materials).

Secondly, CV was used to obtain the oxidation-reduction potentials for the EP, AA, and UA analytes. The CV measurements of EP, UA, and AA were performed in the *E* range from −0.600 V to 0.800 V. These CV measurements were performed using a sweep rate of 50 mV/s. Based on the information obtained (the *E* of the developed peaks), the direction of the potential sweep (anodic or cathodic) was chosen for the SWV measurements. For both CV experiments, the solution was not stirred during the CV measurements.

### 2.4. Square-Wave Voltammetry

SWV was used as the electroanalytical technique for method validation and quantification. SWV was performed using a positive-going square-wave potential sweep in the *E* range from −0.600 V to 0.600 V for the electrochemical oxidation of all analytes and a negative-going square-wave potential sweep from 0.600 V to −0.600 V for the electrochemical reduction of EP. An amplitude of 50 mV, a 4 mV *E* step, and a frequency of 20 Hz were employed in both sweep directions. Before each measurement, a 15 s equilibration time was employed. In all electrochemical experiments, 0.15 M PBS solution (pH 6.5) was used as the supporting electrolyte.

### 2.5. Partial Method Validation

Partial method validation was performed by determining the LOD, LOQ, linear concentration range, precision (as the precision of the method, not as precision of the system), and accuracy. All measurements were performed in triplicate. Within the replicate measurements, the possible presence of outliers was checked by Dixon’s and Grubbs’ statistical tests at 95% confidence [33]. If an outlier was present, this particular value was discarded and not used for the calculation of the average value of the parameters (at least three measurements were performed for all calculations without outliers present).

### 2.6. Real Sample Analysis

All analyte solutions for real sample analysis were prepared in a supporting electrolyte (0.15 M PBS solution at pH 6.5). Before each sample preparation, the PBS was purged with nitrogen for 15 min. All measurements were performed in triplicate.

The EP solution from the EP auto‒injector was determined using SWV in both potential sweep directions, anodic and cathodic. The content of AA in a dietary supplement and the concentration of UA in human urine were determined using SWV in the anodic direction. The urine sample was obtained from one of the authors and used without any sample pretreatment.

To determine the accuracy and precision of the methods, the samples were spiked with a known quantity of the solutions of the analyte’s standard.

## 3. Results and Discussion

### 3.1. Electrochemical Reaction Mechanisms of EP, UA, and AA

Cyclic voltammetry was used to investigate the reduction and oxidation reactions of analytes to obtain information on the electrochemical reaction mechanisms.

**Figure 1:**
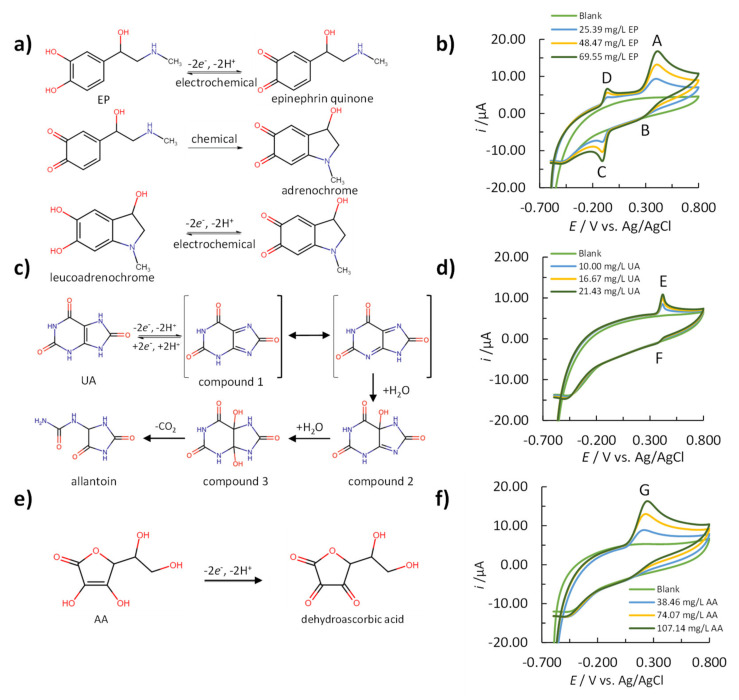
Electrochemical reaction mechanisms for (**a**) EP, (**c**) UA, and (**e**) AA, and measured cyclic voltammograms for (**b**) blank, 25.39 mg/L, 48.47 mg/L, and 69.55 mg/L of EP, (**d**) blank, 10.00 mg/L, 16.67 mg/L, and 21.43 mg/L of UA, and (**f**) blank, 38.46 mg/L, 74.07 mg/L, and 107.14 mg/L of AA in 0.15 M PBS (a potential sweep rate of 50.00 mV/s).

Figure 1b shows the cyclic voltammogram for EP, while its proposed reaction mechanism is presented in Figure 1a. The anodic peak (peak A in Figure 1b) corresponds to the electrochemical oxidation of EP to epinephrine quinone, exchanging two electrons and two protons. The cathodic peak (peak B in Figure 1b) corresponds to the reduction of epinephrine quinone to EP. Peaks C and D demonstrate the reversible electrochemical reaction of the reduction of adrenochrome to leukoadrenochrome (peak C, Figure 1b) followed by its regeneration, while sweeping in the anodic direction (peak D, Figure 1b) [34]. Since EP produces an electrochemical response by sweeping the *E* in both directions (anodic and cathodic), two different methods for its detection were developed and validated using SWV. The peaks selected for EP determination were peaks A and C using anodic and cathodic sweeps, respectively. The terms EP (anodic sweep) and EP (cathodic sweep) are used hereinafter to describe these methods.

In an aqueous medium, UA can be irreversibly oxidized to allantoin. The proposed mechanism of this reaction is presented in Figure 1c. UA is first oxidized to diimine (compound 1, Figure 1c), followed by a nucleophilic attack of water, resulting in imine alcohol (compound 2, Figure 1c). After the addition of the second water molecule, UA-4,5-diol is formed (compound 3, Figure 1c). At neutral pH, compound 3 decomposes into allantoin and CO_2_ [35]. The cyclic voltammogram of UA (Figure 1d) shows a well-defined anodic peak (peak E), which corresponds to the electrochemical oxidation of UA to compound 1. Compound 1 further reacts with water, generating a very poorly defined cathodic peak (peak F). Based on that, the signal used for the development and validation of the electroanalytical method using SWV was the one that developed during the anodic sweep (peak E, Figure 1d).

Electrochemical determination of AA is possible due to its electrochemical oxidation to dehydroascorbic acid [36]. The proposed mechanism is shown in Figure 1e. The cyclic voltammogram in Figure 1f shows a defined anodic peak (peak G). Since no peak is formed by potential sweeping in the cathodic direction, the method for the determination of AA was developed and validated using SWV only in the anodic direction.

### 3.2. Partial Method Validation

#### 3.2.1. LOD and LOQ Determination

The obtained LOD and LOQ values for all 4 methods are presented in Table 1 (the highest LOD and LOQ values out of the triplicate measurements are reported). The obtained LOD and LOQ values are comparable to those reported previously using HPLC, e.g., it was reported that the LOD and LOQ for EP tartrate using HPLC were 0.1 μg/mL and 0.3 μg/mL, respectively [37]. Moreover, the reported LOD and LOQ for AA using HPLC were 1.2 × 10^−3^ g/L and 3.4 × 10^−3^ g/L, respectively [38]. Furthermore, the reported LOD and LOQ for UA using HPLC were 0.30 μg/mL and 0.89 μg/mL, respectively [39]. Therefore, the electroanalytical methods presented in this work are competitive with HPLC for the analysis of EP, AA, and UA in terms of LOD and LOQ. An explanation about the procedure for LOD and the LOQ determination is given in the Supplementary Material.

#### 3.2.2. Linearity

The data for the determination of the linear concentration range were obtained by pipetting increasing amounts of solution of the diluted analyte standard into the electrochemical cell and measuring the current response in voltammograms. The response Δ*i*_p_ (the peak height) vs. analyte concentration was plotted and the linear concentration range was determined using linear least squares regression (Δ*i*_p_ = *b*_1_·*γ* + *b*_0_, where *b*_1_ is the slope, *b*_0_ is the intercept and *γ* is the analyte mass concentration). Linearity was (initially) confirmed if the square of the correlation coefficient (*R*^2^) was ≥0.9950 and the quality coefficient (*QC*) was ≤5.00%. This procedure was employed only for the initial linearity determination, because (as explained in detail below) the values of *R*^2^ and *QC* alone are not enough to test the model fit for the performance of analytical procedures [26,40]. It is also mandatory to evaluate the model fit by checking the data for possible heteroscedasticity. The most common occurrence of heteroscedasticity is an increase in variance as a function of analyte concentration. A study of the heteroscedasticity of the data of the replicate measurements at a single concentration point for the design of calibration curves was performed using a residuals analysis, Hartley’s test, Bartlett’s test, and Cochran’s C test. If the residuals ($e_{i}$) are not randomly distributed around the concentration axis, that indicates the heteroscedastic behavior of the experimental data [33]. The residuals were calculated using Equation (S1) in the Supplementary Material.

Moreover, the normality of the data set for each calibration curve was checked with the Q-Q plot and K-S statistical tests (the normal distribution of all sets of data was confirmed in every case; see Figure S2).

**Figure 2:**
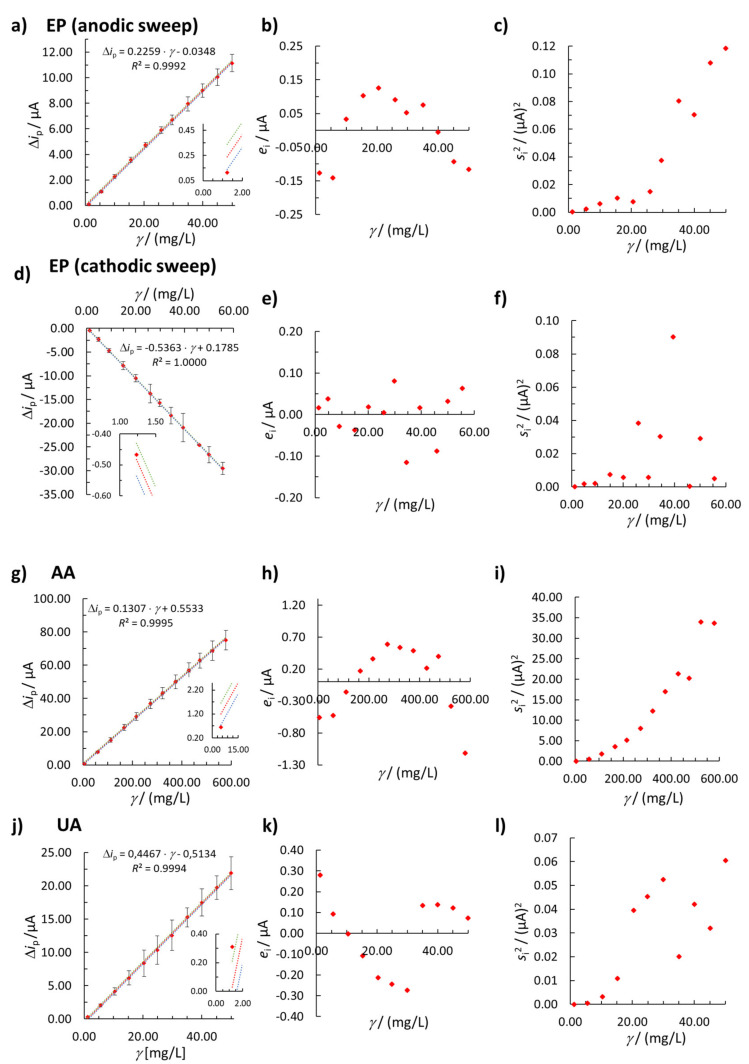
Linear concentration ranges for (**a**) EP (anodic sweep), (**d**) EP (cathodic sweep), (**g**) AA, and (**j**) UA (the error bars in Figure 2a,d,g,j represent standard deviations–for better visual representation, the standard deviation values for EP (anodic sweep) were multiplied by a factor 2, while the standard deviation values for EP (cathodic sweep) and UA were multiplied by a factor of 10, whereas no multiplication was performed for AA). The insert in Figure 2a,d,g,j shows the first calibration point of the linear concentration range, and the dotted curves represent 95% confidence intervals (upper range green and lower range blue). The plots of residuals vs. concentration are shown in (**b**) for EP (anodic sweep), (**e**) for EP (cathodic sweep), (**h**) for AA, and (**k**) for UA. Plots of variance vs. concentration are shown for (**c**) EP (anodic sweep), (**f**) EP (cathodic sweep), (**i**) AA, and (**l**) UA.

The determined linearities for EP (anodic sweep), EP (cathodic sweep), and UA are all in very similar concentration ranges (see Table 2 and Figure 2a,d,j). The upper limit of the UA linear concentration range at 50.00 mg/L is caused by the limited solubility of UA in water. The method for the determination of AA (Figure 2g) shows a linear response in a concentration range from 4.98 mg/L to 578.95 mg/L, which is approximately 10-times wider than the concentration ranges obtained by the other three electroanalytical methods reported herein.

Table 2 shows that all of the methods exhibit high *R*^2^ values (from 0.9992 to 1.0000) and low *QC* values, ranging from 0.40% to 1.78%. On the other hand, the residual plots for EP (anodic sweep, Figure 2b), AA (Figure 2h), and UA (Figure 2k) show that the residuals are not randomly distributed around the concentration axis.

Furthermore, with a change in the concentration of the analytes the standard deviations and variances are not statistically the same (as explained below), which is shown in Figure 2a,d,g,j as the error bars (representing standard deviations) and in Figure 2c,f,i,l as a plot of variance vs. analyte concentration.

The three statistical tests of heteroscedasticity (Hartley’s, Bartlett’s, and Cochran’s C tests) also confirmed the heteroscedastic behavior of the data for EP (cathodic sweep), confirming the unsuitability of the unweighted regression model. On the other hand, for EP (anodic sweep), AA, and UA the assumption of heteroscedasticity was rejected only by Cochran’s C statistical test. However, the residual analysis, Hartley’s test, and Bartlett’s test showed that the unweighted linear regression is not suitable for the quantification of EP (anodic sweep), AA, and UA (Table 2).

Based on the above given facts, the unweighted linear regression model is not appropriate for analysis of these compounds and a weighted linear regression model needs to be taken into account. The latter approach was used for all four sets of analytical data (i.e., EP (anodic sweep), EP (cathodic sweep), AA, and UA) and is presented below.

#### 3.2.3. Weighted Linear Regression

When experimental data follow heteroscedastic behavior, there is a need for a new calibration model that better defines the relationship between the response and the analyte concentration. In order to improve the unsuitable unweighted linear calibration model in the present case, one can choose a different model (such as a quadratic or polynomial model) or use a narrower linear concentration range. However, the aim of this study was not to use a different model (non-linear) or to narrow the linear calibration range, which would make the analytical method less practical for real sample analysis. In order to overcome this issue, a weighted linear regression was employed.

When the data of the analytical response at different calibration points are heteroscedastic, the variances of the replicate measurements are statistically different across the calibration range. The solution to such occurrence is frequently the use of a weighted linear regression to minimize the greater influence of higher concentrations on the regression by putting greater weight on the values with the smallest error and vice versa [41]. The corresponding weighting factors ($w_{i}$) can be calculated from the inverse of the variance obtained by replicate measurements of the response at a certain calibration point i [42], using Equation (S2) in the Supplementary Materials.

However, the use of $w_{i}$ (given in Equation (S2)) is usually impractical because it requires at least three replicate measurements at every concentration level, which extends the analysis time and increases analysis costs. An alternative is the use of an empirical weighting factor ($w_{j}$) based on the x-axis variable (i.e., *γ* in the present case) or the y-axis variable (the response, which is the peak height in the present case), which can be used to provide a simple approximation of variance. The most widely used of such factors are $w_{j}=$ $\frac{1}{x_{j}^{0.5}}\;\mathrm{or}\;\frac{1}{x_{j}}\;\mathrm{or}\;\frac{1}{x_{j}^{2}}\;\mathrm{or}\;\frac{1}{y_{j}^{0.5}}\;\mathrm{or}\;\frac{1}{y_{j}}$ or $\frac{1}{y_{j}^{2}}$ [43]. The choice of the best $w_{j}$ depends on the relative error percentage (RE) calculated by Equation (S3) in the Supplementary Material for a certain calibration point i.

The best $w_{j}$ is determined by plotting RE vs. *γ* and by calculating the sum of the absolute RE values for all calibration points (|ΣRE|). By calculating the |ΣRE|, the effectiveness of the various weighting schemes can be compared to determine which $w_{j}$ provides the lowest method error, i.e., the lowest |ΣRE|. According to the US Food and Drug Administration, an analyst should use the simplest model that adequately describes the concentration-response relationship (using appropriate weighting), so the correct choice is to employ the lowest amount of weighting that gives the smallest |ΣRE| [44].

**Figure 3:**
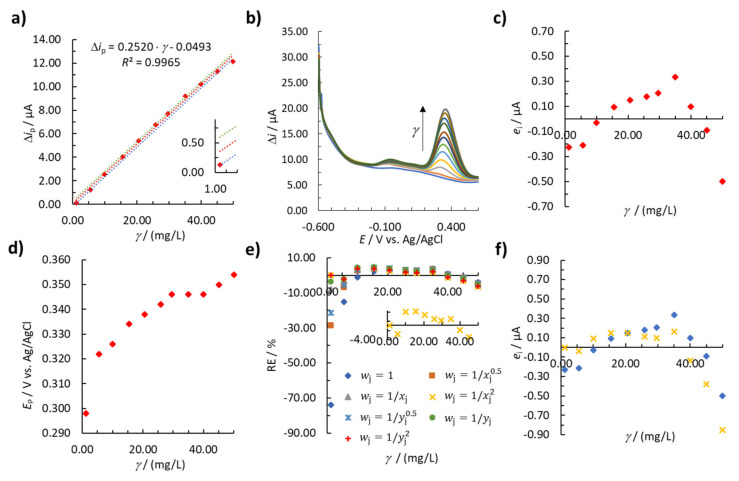
(**a**) The linear calibration curve for EP (anodic sweep) using weighted regression analysis, (**b**) voltammograms measured when the linear calibration curve was constructed, (**c**) plot of residuals vs. concentration, (**d**) the change in E_P_ with increasing EP concentration, (**e**) RE vs. γ for the unweighted and weighted regression models, and (**f**) a comparison of residuals for the unweighted (blue) and the best weighted regression model, i.e., Model 4 (yellow). The insert in Figure 3a represents the first point of the linear concentration range, and 95% confidence intervals (green and blue lines).

In the next step in the development of the analytical methods, linear calibration curves were determined (first by employing the unweighted regression model) using one measurement for every calibration point. Again, the normality of the data, for all four analytical methods, was confirmed by the Q-Q plot and K-S statistical tests (see Figure S3 in the Supplementary Material). As expected, the residual plot for EP (anodic sweep) in Figure 3c shows a non-random distribution and thus heteroscedastic behavior, justifying the application of a weighted linear regression (the same was reported above in Figure 2b using the average value of the method response based on the three replicate measurements). The weighted regression model parameters (*b*_1_, *b*_0_, and *R*^2^) were calculated for each of the above mentioned $w_{j}$ according to Equations (S4)–(S6), respectively (see Supplementary Material). The results obtained according to this procedure for EP (anodic sweep) are shown in Table 3.

The weighted regression models were compared with the unweighted regression model ($w_{j}$ = 1) by calculating the |ΣRE|. Model 4 ($w_{j}$ = $\frac{1}{x_{j}^{2}}$) gives the smallest |ΣRE| and the best distribution (the smallest values for certain calibration points in general) of RE around the concentration axis (Figure 3e) compared to the other generated weighted regression models shown in Table 3.

For the best weighted regression model, a residual analysis was performed and compared with the residual analysis of the unweighted regression model (Figure 3f). For the lowest and second lowest calibration points, the residuals are significantly smaller using Model 4 compared to Model 1 (Figure 3f). Therefore, Model 4 produces a smaller error at lower EP concentrations and therefore makes the analytical method more useful. Considering the above, the best $w_{j}$ was $\frac{1}{x_{j}^{2}}$ and Model 4 was used to validate the method’s accuracy and precision, as presented below.

The measured voltammograms for EP (anodic sweep) are shown in Figure 3b. The peak’s potential (*E*_P_) is in the *E* range from 0.298 V to 0.354 V. *E*_P_ is shifted toward more positive *E* by increasing the EP concentration from 1.22 mg/L to 49.97 mg/L (Figure 3d).

The same approach was used to determine the best $w_{j}$ and weighted regression model for EP (cathodic sweep), AA, and UA determination. The best determined $w_{j}$ (i.e., producing the lowest ∑|RE| among all $w_{j}\;$ tested) and corresponding *b*_1_, *b*_0_, and *R*^2^ determined for EP (cathodic sweep), AA, and UA are shown in Table 4. The best obtained $w_{j}$ for the determination of EP (cathodic sweep) and AA was determined to be $\frac{1}{y_{j}^{2}}$, whereas the best $w_{j}$ for the determination of UA was $\frac{1}{x_{j}^{2}}$. On that basis, these newly determined weighted regression models were used to validate the methods’ accuracy and precision, as presented below.

#### 3.2.4. Accuracy and Precision of the Method

Both the accuracy and precision of the methods were evaluated at the low, middle, and high concentration levels of the linear concentration ranges (which were reported in Table 2) by spiking a 0.15 M PBS solution with a solution of analyte standard. The accuracy and precision were evaluated in terms of recovery and relative standard deviation (RSD), respectively (RSD and recovery validation criteria are explained in the Supplementary Material). The concentration of the analyte in the real samples was determined using the best weighted regression model (the calibration curve reported in Table 3 and Table 4). The use of a calibration curve for quantification purposes is not frequently employed in electroanalysis and presents a time-saving alternative to the typically used multiple standard addition methodology.

The obtained results are presented in Table 5 and show that the measurements for EP and AA are accurate and precise at all three concentration levels. The method for UA is precise at all levels (the average RSD values are well below 20.00%). However, the average recovery value at a low UA concentration level is 78.58%, which is outside the recovery limits (but close to the lower limit of 80.00%). It therefore can be concluded that the method for the determination of UA is accurate and precise only at the middle and upper levels.

### 3.3. Real Sample Analysis

The applicability of the newly developed and validated methods based on weighted regression models was tested by determining the concentration of EP in an EP auto-injector, AA in a dietary supplement, and UA in human urine. Concentration determination was carried out using the calibration curve methodology as presented above. The content of the sample and the method’s accuracy and precision were determined using the best weighted linear regression model as reported above.

The obtained results, presented in Table 6, show that all the methods for the real sample analysis have satisfactory accuracy (recovery was within the 80.00–120.00% recovery interval). Moreover, all methods have satisfactory precision (the RSD were well below 20.00%). Moreover, the determined concentration of UA in human urine (provided by one of the authors) corresponds to the expected concentration range reported in the literature [45]. It has to be pointed out that no sample pretreatment was carried out prior to urine analysis. Based on the above-given results, it can be concluded that all of the developed methods are suitable for real sample analysis. Voltammograms for the real samples are included in the Supplementary Material (see Figure S4).

## 4. Conclusions

This study presents the development of electrochemical methods based on square-wave voltammetry (SWV) for the quantification of three different analytes, namely epinephrine (EP), ascorbic acid (AA), and uric acid (UA). Weighted linear regression models were employed to validate these methods.

The methods for the determination of AA and UA were developed using an anodic potential sweep. Since EP gave a response when the potential was swept in both directions (anodic and cathodic potential sweeps), two different methods for its determination were developed. Method validation was performed by determining the limit of detection (LOD), limit of quantification (LOQ), linearity, accuracy, and precision. The normal distribution of all data sets was confirmed using the quantile-quantile (Q-Q) plot and Kolmogorov-Smirnov (K-S) statistical tests.

The determined LODs were 0.62 mg/L for EP (using an anodic potential sweep), 0.25 mg/L for EP (using a cathodic potential sweep), 0.50 mg/L for AA, and 0.62 mg/L for UA. The corresponding LOQ values were determined to be 1.10 mg/L (for EP using an anodic potential sweep), 0.49 mg/L (for EP using a cathodic potential sweep), 1.98 mg/L (for AA), and 1.22 mg/L (for UA). AA showed the widest linear concentration range, i.e., from 4.98 mg/L to 578.95 mg/L, whereas the linear concentration ranges for the other three methods were 1.22‒49.97 mg/L (for EP using an anodic potential sweep), 1.23‒55.52 mg/L (for EP using a cathodic potential sweep), and 1.22‒50.00 mg/L (for UA).

Homoscedasticity is an important assumption that must not be neglected in the calibration process. It was found that the variances of the y-values (the response of the analytical methods) are not statistically the same at different analyte concentrations for the four data sets, indicating heteroscedastic behavior. This assumption was confirmed using Hartley’s test, Bartlett’s test, Cochran’s C test, and the analysis of residuals. The regression models were then improved using a weighted linear regression. The criteria for the selection of the best empirical weighting factors were the minimum sum of the absolute relative error (RE) values (|ΣRE|) and the distribution of the RE values around the concentration axis. The best weighted regression models for EP (using an anodic potential sweep) and UA were weighted by an empirical weighting factor of $\frac{1}{x_{j}^{2}}$, while the best results for the EP (using a cathodic potential sweep) method and AA method were obtained when the regression models were weighted by an empirical weighting factor of $\frac{1}{y_{j}^{2}}$. The selected weighted regression models were used for the determination of the accuracy and precision of the methods. The developed and validated methods were successfully used for the analysis of real samples by determining EP in an EP auto-injector, AA in a dietary supplement, and UA in human urine. The recovery and relative standard deviation values for these four methods were in the range of 95.21–113.23% and 0.85–7.98%, respectively.

By employing SWV, the voltammogram is obtained in just 40 s in aqueous media, making the presented methods for the quantification of EP, AA, and UA an advantageous alternative to chromatographic techniques.

## Figures and Tables

**Table 1:** Summary of LOD and LOQ values determined by each SWV method along with the obtained S/N values.

	LOD/(mg/L)	S/N	LOQ/(mg/L)	S/N
EP (anodic sweep)	0.62	4.78	1.10	11.48
EP (cathodic sweep)	0.25	3.99	0.49	10.23
AA	0.50	5.72	1.98	11.67
UA	0.62	3.56	1.22	10.85

**Table 2:** A summary of the linear concentration range parameters using the unweighted linear regression model, normality test results, and homoscedasticity test results.

Analyte	EP (Anodic Sweep)	EP (Cathodic Sweep)	AA	UA
Linear concentration range/(mg/L)	1.22–49.97	1.23–55.52	4.98–578.95	1.22–50.00
*b*_1_	0.2259	−0.5363	0.1307	0.4467
*b*_0_	0.0348	0.1785	0.5533	0.5134
*QC/*%	1.78	0.40	1.39	1.70
*R* ^2^	0.9992	1.0000	0.9995	0.9994
Normality				
Q-Q plot	passed	passed	passed	passed
K-S test	passed	passed	passed	passed
Homoscedasticity				
Residual plot	not random	random	not random	not random
Hartley’s test	not passed	not passed	not passed	not passed
Bartlett’s test	not passed	not passed	not passed	not passed
Cochran’s C test	passed	not passed	passed	passed

**Table 3:** The regression parameters of the unweighted regression model ($w_{j}$ = 1) and the regression parameters of the weighted analytical models for EP (anodic sweep) with the corresponding sums of the relative errors (∑|RE|).

	Model No. for (Anodic Sweep)	$w_{j}$	*b*_1_	*b*_0_	*R* ^2^	∑|RE|/%
	1 (unweighted regression)	1	0.2520	0.0493	0.9965	110.57
**weighted regression models**	2	$\frac{1}{x_{j}^{0.5}}$	0.2570	−0.0941	0.9977	60.99
	3	$\frac{1}{x_{j}}$	0.2601	−0.1621	0.9984	38.63
	4	$\frac{1}{x_{j}^{2}}$	0.2638	−0.1919	0.9988	28.16
	5	$\frac{1}{y_{j}^{0.5}}$	0.2573	−0.1168	0.9979	54.34
	6	$\frac{1}{y_{j}}$	0.2603	−0.1761	0.9985	34.46
	7	$\frac{1}{y_{j}^{2}}$	0.2633	−0.1917	0.9988	28.62

**Table 4:** A comparison of the unweighted (Model 1, $w_{j}\;$ = 1) and best weighted regression models with the corresponding regression parameters and the sum of the absolute RE values (∑|RE|) obtained for EP (cathodic sweep), AA, and UA analytes.

Analyte	Model No.	$w_{j}$	*b*_1_	*b*_0_	*R* ^2^	∑|RE|/%
EP (cathodic sweep)	1	1	−0.4797	0.1153	0.9994	12.18
	7	$\frac{1}{y_{j}^{2}}$	−0.4792	0.1084	0.9999	11.05
AA	1	1	0.1367	1.1455	0.9988	213.62
	7	$\frac{1}{y_{j}^{2}}$	0.1425	−0.1470	0.9993	25.03
UA	1	1	0.4373	−0.3546	0.9999	32.90
	4	$\frac{1}{x_{j}^{2}}$	0.4282	−0.2078	0.9995	17.34

**Table 5:** Average recoveries and RSD values using the weighted regression model.

	*γ* /(mg/L) (Spiked)	*γ* /(mg/L) (Determined)	Recovery/%	RSD/%
EP (anodic sweep)	4.54	4.93	108.56	0.08
	27.76	27.60	99.43	5.23
	47.59	45.99	96.64	13.64
EP (cathodic sweep)	2.44	2.37	97.30	3.02
	27.26	26.21	96.17	2.80
	52.91	50.56	95.55	0.57
AA	24.39	21.26	87.16	3.15
	298.25	290.48	97.39	1.27
	574.47	579.31	100.84	2.88
UA	2.38	1.87	78.58	5.54
	25.00	23.14	92.57	3.80
	47.96	41.09	85.68	6.55

**Table 6:** Results of real sample analysis for all 4 methods using the weighted regression model presented in Table 3 and Table 4.

	^a^*γ/*(mg/L) (Initially Found)	^b^*γ/*(mg/L) (Spiked)	^c^*γ/*(mg/L) (Found)	Recovery/%	RSD/%	Content of the Sample/(mg/L)
EP (anodic)	12.50	10.98	24.09	105.50	1.83	483.67
EP (cathodic)	6.68	11.59	17.72	95.21	0.85	452.08
AA	200.44	215.69	423.08	103.22	2.86	255.57
UA	3.46	25.00	31.77	113.23	7.98	345.41

^a^ Determined *γ* of the diluted sample. ^b^ *γ* of the solution of the diluted analyte standard that was used to spike the real sample. ^c^ Determined *γ* after spiking the sample with the solution of the diluted analyte standard.

## Supplementary Materials

The following are available online at https://www.mdpi.com/1424-8220/20/24/7056/s1, Figure S1: A glassy carbon working electrode immersed in 1.0 M KCl solution containing 10 mM K_3_[Fe(CN)_6_]; (a) CV voltammogram at different sweep rates (ν), (b) *i*_pa_ vs. $\sqrt{\nu }$, and (c) *i*_pc_ vs. $\sqrt{\nu }$. Figure S2: (a,c,e,g) Q-Q plots and (b,d,f,h) K-S statistical tests confirming the normal distribution of the data for the first set of calibration curves (the average response out of three replicate measurements at every calibration point) for (a,b) EP (anodic sweep), (c,d) EP (cathodic sweep), (e,f) AA, and (g,h) UA determination. The parameter z_theoretical_ represents the z-value of the standard normal distribution, z_actual_ is the actual z-value calculated based on the experimental data. F_O_ and F_E_ stand for the observed and expected frequency, respectively. F_Ok_ and F_Ok-1_ represent F_O_ for the k/n and k-1/n (k = 1, 2, …, n) calibration points, respectively. Figure S3: (a,c,e,g) Q-Q plots and (b,d,f,h) K-S statistical tests confirming the normal distribution of the data for the second obtained set of calibration curves (one measurement for every calibration point) that were used for the weighted linear regression; for (a,b) EP (anodic sweep), (c,d) EP (cathodic sweep), (e,f) AA, and (g,h) UA determination. Figure S4: Voltammograms for the real samples measured using SWV in 0.15 M PBS; (a) EP from epinephrine autoinjector (anodic sweep), (b) EP from epinephrine autoinjector (cathodic sweep), (c) AA from a nutrition supplement, and (d) UA from a human urine sample.
