# Supplementary material for: An Advanced Statistical Approach Using Weighted Linear Regression in Electroanalytical Method Development for Epinephrine, Uric Acid and Ascorbic Acid Determination
Source: Sensors (Basel). 2020 Dec 9;20(24):7056. doi: 10.3390/s20247056 (PMC7763546; doi:10.3390/s20247056)
Supplement: Supplementary file 1 [file sensors-20-07056-s001.pdf]

SUPPLEMENTARY MATERIAL

An advanced statistical approach using weighted linear regression in electroanalytical method development for epinephrine, uric acid, and ascorbic acid determination

David Majer[1], Tinkara Mastnak[1], Matjaž Finšgar[*,1]

[1]*University of Maribor, Faculty of Chemistry and Chemical Engineering, Smetanova ulica 17, 2000 Maribor, Slovenia*

1. The reversibility of the potassium hexacyanoferrate system

The reversibility of the diffusion-controlled potassium hexacyanoferrate ($K_3[Fe(CN)_6]$) system was checked one time per day before the electroanalytical measurements in order to determine the appropriateness of the glassy carbon working electrode. In this work, four criteria were investigated to check if the $K_3[Fe(CN)_6]$ oxidation/reduction reaction is reversible and diffusion-controlled: i) the difference between the anodic peak potential (E_{pa}) and the cathodic peak potential (E_{pc}), ΔE_p, should be 59.00 mV (for one-electron transfer redox reaction), ii) E_{pa} and E_{pc} should not change with increasing potential sweep, iii) the absolute value of the anodic peak current (i_{pa}) and cathodic peak current (i_{pc}) ratio should be equal to 1, and iv) i_{pa} and i_{pc} should change linearly with $\sqrt{\nu}$ [1]. For the $K_3[Fe(CN)_6]$ diffusion-controlled reversible system, the theoretical value of ΔE_p is 59.00 mV, as the number of exchanged electrons in the redox reaction is 1. At a potential sweep rate (ν) of 10.00 mV/s, the experimentally obtained values ΔE_p were between 72.00 and 76.00 mV. The potential difference then increased with increasing ν. Figure S1 shows a slight shift of E_{pa} towards more positive potentials and of E_{pc} towards more negative potentials with increasing ν. The reason for the E_{pa} and E_{pc} shifts and consequently the larger potential difference than the theoretical can be explained with the *iR*-ohmic drop (where *i* is current and *R* is the solution resistance). Thus, the potential resulting from the *iR*-drop must be added to the applied potential on the working electrode (in the case of an anodic ν). This effect was minimized by installing working and reference electrodes at the closest distance possible. Considering the above mentioned, the potential shift is not significant. The third criterion was met as the ratios of i_{pa} and i_{pc} were close to 1. Finally, i_{pa} and i_{pc} changed linearly with increasing $\sqrt{\nu}$ as the R^2 were higher than 0.9950 and therefore the fourth criterion was met. Furthermore, the diffusion coefficients were calculated using the Randles-Sevčink equation and compared with values reported in the literature. The diffusion coefficients were in the same order of magnitude as the reported value, which is a good indicator of a properly working electrode. We can conclude that despite small

[*] Corresponding author
Address: University of Maribor, Faculty of Chemistry and Chemical Engineering, Smetanova ulica 17, 2000 Maribor, Slovenia
e-mail: matjaz.finsgar@um.si; *phone*: +386 2 2294 447

deviations from the ideal required conditions, the glassy carbon electrode fulfils all four criteria and can be used for further electroanalysis.

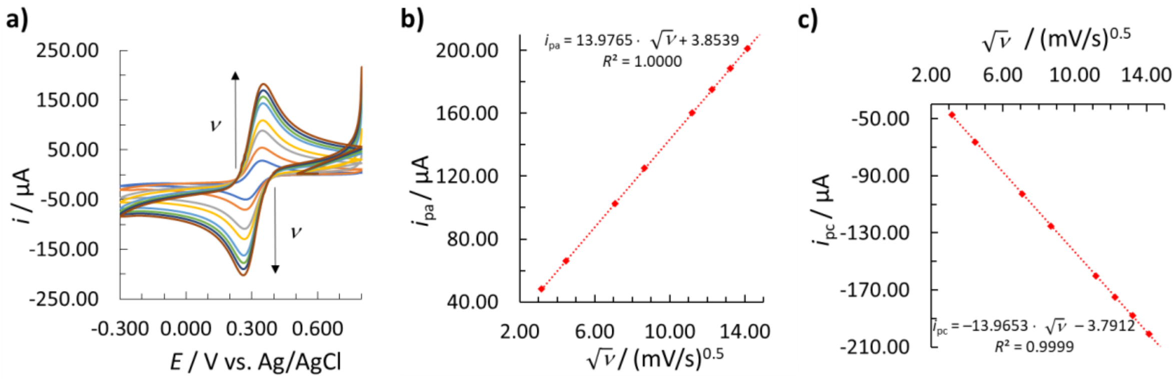

Figure S1: A glassy carbon working electrode immersed in 1.0 M KCl solution containing 10 mM $K_3[Fe(CN)_6]$; a) CV voltammogram at different sweep rates (ν), b) i_{pa} vs. $\sqrt{\nu}$, and c) i_{pc} vs. $\sqrt{\nu}$.

2. LOD and LOQ determination

The LOD and LOQ values were determined experimentally based on the signal to noise (S/N) ratio, where S represents the analyte's peak height and N represents the background noise (determined as the difference between the highest and lowest points in the background contribution at the more positive or more negative *E* side of the analyte's peak). The S/N ratio was obtained by performing SWV measurements by successively injecting diluted solutions of the analyte's standard and measuring the current response. The criterion for LOD was S/N ≥ 3.00 (but close to 3.00 and lower than 10.00), and the criterion for LOQ was S/N ≥ 10.00 (but close to 10.00)[2].

3. Linearity

$$e_{\mathrm{i}} = (\Delta i_{\mathrm{measured}})_{\mathrm{i}} - (\Delta i_{\mathrm{model}})_{\mathrm{i}} \qquad \text{(Equation S1)}$$

Where $(\Delta i_{\mathrm{measured}})_{\mathrm{i}}$ is the measured current at the ith calibration point (peak height) and $(\Delta i_{\mathrm{model}})_{\mathrm{i}}$ is the corresponding interpolated signal from the obtained regression equation.

3.1 Weighted Linear Regression

$$w_{\mathrm{i}} = \frac{n \cdot s_{\mathrm{i}}^{-2}}{\sum_{\mathrm{i}=1}^{n} s_{\mathrm{i}}^{-2}} \qquad \text{(Equation S2)}$$

Where s_{i}^{-2} is the square of the inverse of the variance response at the ith calibration point and n is the number of calibration points.

$$\mathrm{RE}\ /\ \% = \frac{(\gamma_{\mathrm{model}})_{\mathrm{i}} - (\gamma_{\mathrm{theoretical}})_{\mathrm{i}}}{(\gamma_{\mathrm{theoretical}})_{\mathrm{i}}} \cdot 100 \qquad \text{(Equation S3)}$$

Where $(\gamma)_{\mathrm{i}}$ is the *g* at a certain calibration point given by the weighted regression model upon response measurement, and $\gamma_{\mathrm{theoretical}}$ is the theoretical *g* of the solution of the diluted analyte standard at a certain calibration point i.

$$b_1 = \frac{n \cdot \sum_{\mathrm{j}=1}^{n} w_{\mathrm{j}} x_{\mathrm{j}} y_{\mathrm{j}} - \sum_{\mathrm{j}=1}^{n} w_{\mathrm{j}} x_{\mathrm{j}} \cdot \sum_{\mathrm{j}=1}^{n} w_{\mathrm{j}} y_{\mathrm{j}}}{n \cdot \sum_{\mathrm{j}=1}^{n} w_{\mathrm{j}} x_{\mathrm{j}}^2 - (\sum_{\mathrm{j}=1}^{n} w_{\mathrm{j}} x_{\mathrm{j}})^2} \qquad \text{(Equation S4)}$$

$$b_0 = \frac{\sum_{j=1}^{n} w_j y_j - b_1 \cdot \sum_{j=1}^{n} w_j x_j}{n} \qquad \text{(Equation S5)}$$

Where x_j is the g and y_j is the signal (the response of the analytical method) at a certain concentration point j. The weighted R^2, which describes the statistical relationship between two variables, is calculated by Equation S6.

$$R^2 = \left(\frac{\sum_{j=1}^{n} w_j \cdot \sum_{j=1}^{n} w_j x_j y_j - \sum_{j=1}^{n} w_j x_j \cdot \sum_{j=1}^{n} w_j y_j}{\sqrt{\sum_{j=1}^{n} w_j \cdot \sum_{j=1}^{n} w_j x_j^2 - \left(\sum_{j=1}^{n} w_j x_j\right)^2} \cdot \sqrt{\sum_{j=1}^{n} w_j \cdot \sum_{j=1}^{n} w_j y_j^2 - \left(\sum_{j=1}^{n} w_j y_j\right)^2}} \right)^2 \qquad \text{(Equation S6)}$$

4. Accuracy and Precision of the Method

To test precison and accuracy, new solutions were prepared every time before electroanalytical determination. The recovery was calculated as recovery % = 100.00 · $\gamma_{\text{determined}}/\gamma_{\text{theoretical}}$. The determined concentration ($\gamma_{\text{determined}}$) is obtained using the weighted regression model. The measurement is deemed to be accurate if the recovery is between 80.00% and 120.00%. For the method to be precise, an RSD value of < 20.00% was considered [3, 4]. At least three replicate measurements were performed, and the presence of possible outliers were checked using Dixon's and Grubbs' statistical tests. If an outlier was present, this particular value was discarded and not used for the calculation of the average recoveries and RSD values. In that case, the electrochemical cell was spiked again, and the recovery and RSD values were determined until three measurements were obtained at every tested level without outliers present.

5. Q-Q plots and Kolmogorov Smirnov (K-S) statistical tests

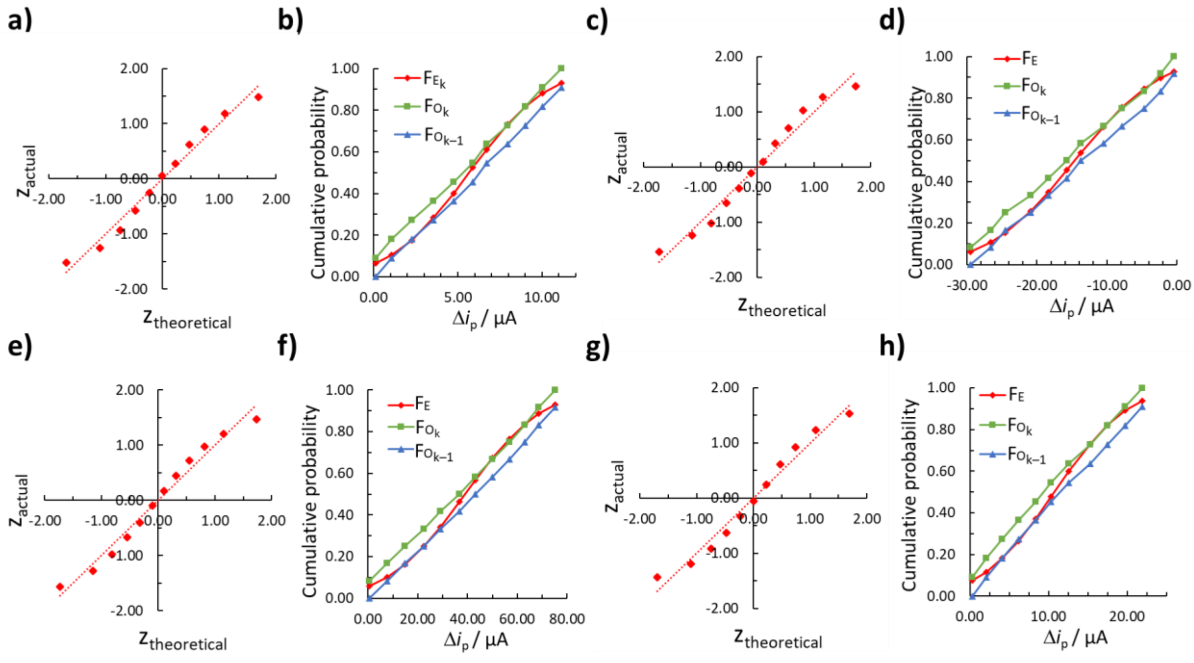

Figure S2: a, c, e, g) Q-Q plots and b, d, f, h) K-S statistical tests confirming the normal distribution of the data for the first set of calibration curves (the average response out of three replicate measurements at every calibration point) for a,b) EP (anodic sweep), c,d) EP (cathodic sweep), e,f) AA, and g,h) UA determination. The parameter $z_{\text{theoretical}}$ represents the z-value of the standard normal distribution, z_{actual} is the actual z-value calculated based on the experimental data. F_O and F_E stand for

the observed and expected frequency, respectively. F_{O_k} and $F_{O_{k-1}}$ represent F_O for the k/n and k–1/n (k = 1, 2, ..., n) calibration points, respectively [5].

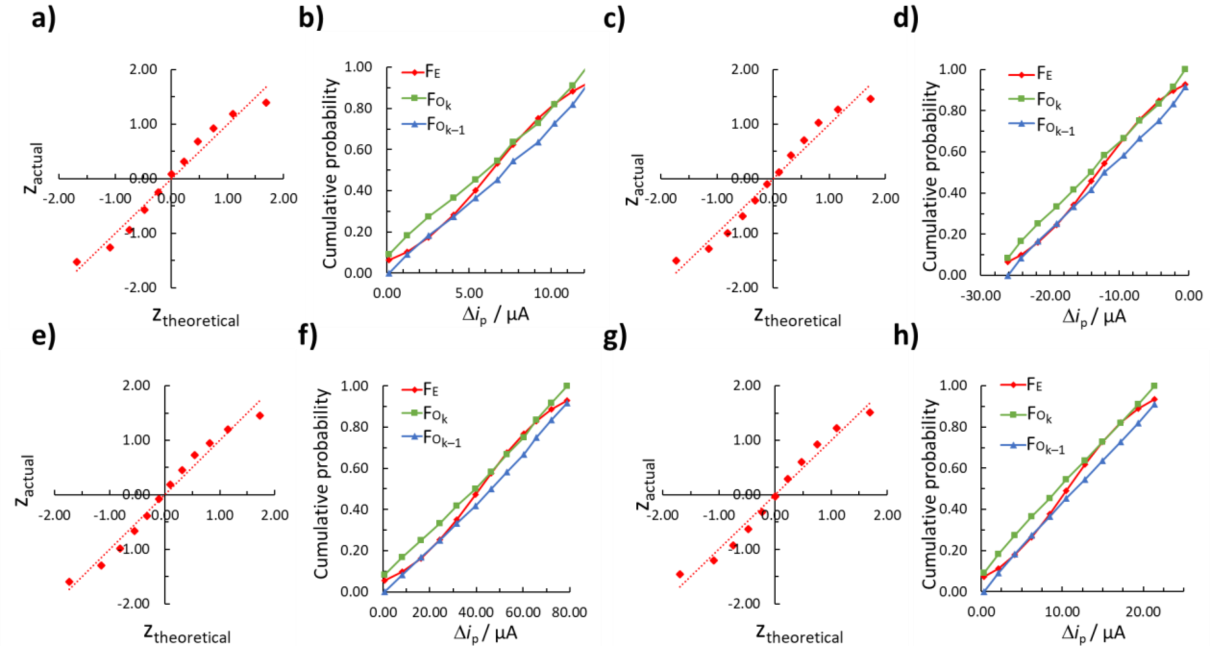

Figure S3: a, c, e, g) Q-Q plots and b, d, f, h) K-S statistical tests confirming the normal distribution of the data for the second obtained set of calibration curves (one measurement for every calibration point) that were used for the weighted linear regression; for a,b) EP (anodic sweep), c,d) EP (cathodic sweep), e,f) AA, and g,h) UA determination.

6. Voltamograms for the real sample analysis

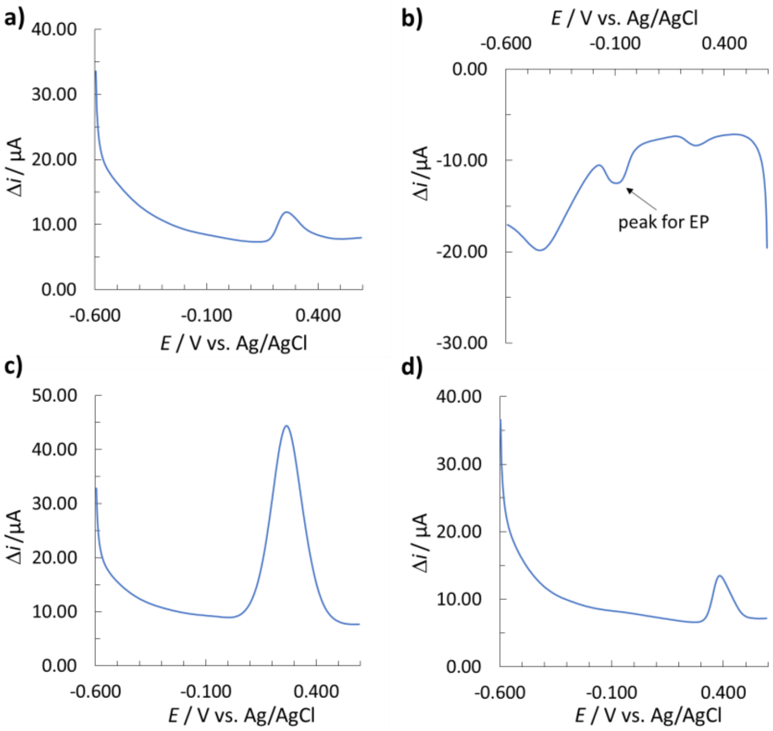

Figure S4: Voltammograms for the real samples measured using SWV in 0.15 M PBS; a) EP from epinephrine autoinjector (anodic sweep), b) EP from epinephrine autoinjector (cathodic sweep), c) AA from a nutrition supplement, and d) UA from a human urine sample.

7. References

1. Matjaz, F., X. Klodian, and O.Ć. Helena, *Cyclic Voltammetry as an Electroanalytical Tool for Analysing the Reaction Mechanisms of Copper in Chloride Solution Containing Different Azole Compounds.* Current Analytical Chemistry, 2020. **16**(4): p. 465-474.
2. *Q2(R1) Validation of Analytical Procedures*, in *ICH Quality Guidelines*. 2017. p. 127-166.
3. *Guidance for the Validation of Analytical Methodology and Calibration of Equipment used for Testing of Illicit Drugs in Seized Materials and Biological Specimens*. 2009, United Nations New York.
4. Laboratory and Scientific Section United Nations Office on Drugs and Crime, V., *Guidance for the Validation of Analytical Methodology and Calibration of Equipment used for Testing of Illicit Drugs in Seized Materials and Biological Specimens, A commitment to quality nd continuous improvement*. 2009, New York: United Nations.
5. Massart, D.L., et al., *Handbook of Chemometrics and Qualimetrics: Part A*. 1997, Amsterdam: Elsevier.
